# Assessing the Prevalence of Orthorexia Nervosa in a Sample of University Students Using Two Different Self-Report Measures

**DOI:** 10.3390/ijerph16142459

**Published:** 2019-07-11

**Authors:** María Laura Parra-Fernández, María Dolores Onieva-Zafra, Elia Fernández-Martínez, Ana Abreu-Sánchez, Juan José Fernández-Muñoz

**Affiliations:** 1 Department of Nursing, Physiotherapy and Occupational Therapy, Faculty of Nursing of Ciudad Real, Universidad de Castilla-La-Mancha, 13071 Ciudad Real, Spain; 2 Department of Nursing, University of Huelva, 21004 Huelva, Spain; 3 Department of Psychology, University Rey Juan Carlos, 28922 Alcorcón, Madrid, Spain

**Keywords:** orthorexia nervosa, ORTO-11-ES, DOS-ES prevalence, university students

## Abstract

In recent decades, orthorexia nervosa (ON) has increased presence in society. It is related with beliefs and attitudes towards eating and is characterized by an obsessive behavior toward heathy eating. The prevalence of ON has been reported by numerous researchers, with rates varying considerably according to the tool used to evaluate the same parameter. The aim of this study was to compare the prevalence of ON in a single population using two different questionnaires. The test for the diagnosis of orthorexia (ORTO-11-ES) assessment tool for orthorexia nervosa and the Düsseldorfer Ortorexie Skala (DOS-ES), constitute brief self-report assessment tools which measure the risk of suffering ON. A sample of 492 students from the University of Castilla la Mancha (Spain) participated in this study, of whom 43.1% were male and 56.9% were female. The findings show that, according to the DOS-ES, only 10.5% of students displayed ON whereas, with the ORTO-11-ES, the prevalence of ON increased to 25.2%. The tendency towards orthorexic behavior is more closely associated with the female gender. The Body Mass Index (BMI) had no influence on the tendency for ON. This study provides valuable information on the usefulness of both questionnaires and the possible limitations associated with the use of these tools in the general population.

## 1. Introduction

Orthorexia nervosa (ON) is a behavior where affected individuals demonstrate an obsession for healthy eating, causing or potentiating damage to the individual on a physical, psychological, and social level. Individuals with ON experience an obsessive–compulsive behavior in the selection, planning, purchase, preparation, and consumption of healthy food, associating healthy properties to certain foods, which may have preventive or healing properties for certain illnesses [1].

Although the scientific community has not yet agreed on definitive diagnostic criteria, some authors have attempted a clarification. Notwithstanding, no consensus has been reached, to date, regarding an absolute definition of ON [1,2,3]. While certain authors have suggested classifying ON as a different and independent disorder, others have related it with eating and obsessive–compulsive disorders, whereas others point to similarities with autism spectrum disorders [4,5,6]. To date, ON is not included with a specific diagnosis in the primary mental health manual, the Statistical Manual of Mental Disorders (DSM-V) [7], nor in the tenth edition of the International Classification of Diseases (ICD-10) [8]. Furthermore, current research in the field of ON has mainly focused on researching the prevalence among different populations, which is variable depending on the respective country, the selected population, and the tool used [9,10,11]. Some authors have attributed these variations in the informed ranges to sociocultural differences between countries [12,13], problems in the formulation of the items in the different tools [14,15], and variability in the cut-off rates in the different adaptations due to differences in language [14,16]. However, what is most questioned, currently, is the need to differentiate individuals who eat healthy foods in a normalized manner from those who truly have a problem with this type of eating, taking it to unhealthy extremes [1,17].

What the scientific community has clarified is the need to use reliable and validated tools, as these are essential for measuring the prevalence rate, estimating risk indexes, and formulating public health recommendations for specific groups. To identify people with ON, several questionnaires have been published, to date. The Orthorexia Self-Test was developed by Bratman and the Bratman Orthorexia Scale (BOT) was the first scale used to measure the risk of ON [18]. Thereafter, and based on this scale, the test for the diagnosis of orthorexia (ORTO-15) [9] was created and validated by Donini et al. in an Italian population. This study reported the first prevalence rates which equaled 6.8% of individuals at risk of suffering ON. To date, this tool, in its various versions, is the most commonly used by the scientific community. The ORTO-15 has been validated and adapted to different languages revealing different factor-structure models [10,16,19,20,21]. In these studies, the reported prevalence varies, from rates considering 17% of students at risk of ON using the ORTO-11-ES [22] to up to 36.9% in Turkey based on the ORTO-11 [23]. The most recent tool is the Düsseldorf Orthorexia Scale (DOS) [11] created by Barthels et al. in a German population, where the psychometric aspect showed good results regarding reliability and validity measurements. This scale has also been recently validated in the United States, China, and Spain [13,24,25]. In Germany, Depa et al. [26] used the DOS and reported prevalence rates of 3.3% of people with ON and 9.0% of people at risk of developing ON among German university students, while in the EEUU the prevalence rate was 8% of ON and 12.4% of people were at risk [24]. In Spain, we found that the adaptation and validation of both scales performed by Parra et al. [14,27], had good psychometric properties.

If we compare the results of the prevalence reported in other countries using both questionnaires, major differences can be observed in the informed ranges. In the US, Dunn et al. found a prevalence rate of 71% with the ORTO-15 [28], whereas another US study performed by Chard et al. using the DOS scale found a prevalence of 8.0% of students with ON, with an additional 12.4% considered at risk of developing ON [24]. Likewise, in Germany, variable prevalence rates were reported, ranging from 69.7% with the ORTO to 3.3% with the DOS.

Most research to date has been performed on university students, including the validation studies concerning these tools. Indeed, university students are a homogeneous sample that can be easily accessed by researchers. Additionally, university students begin their adult life away from their home, having to adopt and/or adapt to changes which include new lifestyles and which in many cases can have different influences, placing this population at risk of suffering different disorders related to their lifestyle. A number of studies suggest that a curriculum specifically directed at improving students’ knowledge of nutrition could lead to a greater tendency to develop ON [16,29,30]. No previous research has investigated the prevalence in the same population using both questionnaires. The hypothesis of this study is that the prevalence of ON detected in a population of Spanish university students differs according to whether we apply the ORTHO-11-ES [14] or the DOS-ES [25] tools.

## 2. Materials and Methods

### 2.1. Ethical Approval

The study protocol was approved by the ethics committee of the General University Hospital of Ciudad Real (Spain) (number C-240).

### 2.2. Participants and Procedure

The participants were recruited from the Ciudad Real campus within the Castilla La Mancha University in Spain. The selected students were studying for degrees in health science, engineering, or architecture. The inclusion criteria consisted of participants who were enrolled at the university in the year 2017/2018. There were no exclusion criteria. The data were gathered via the internet using the JotForm platform and the students voluntarily enrolled in the study. In total, 640 students were asked to complete the online questionnaire developed by the authors, with a response rate of 70.28%. The analyses were focused on a sample of 492 students. In total, 43.1% students were male and 56.9% were female, with an age range from 18 to 44 years and an average age of 19.97 years (SD = 3.03). The mean Body Mass Index was 22.64 (SD = 6.59). The students came from two university faculties, health sciences (63.8%) and engineering (36.2%).

### 2.3. Instruments

#### 2.3.1. Demographic Information

The sociodemographic forms gathered information on the age, gender, height, and weight of participants. The BMI of each participant was calculated based on the self-reported height and weight.

#### 2.3.2. The Spanish Version of the Düsseldorfer Orthorexie Skala (DOS-ES)

The DOS questionnaire was originally created by Barthels et al. [24]. This scale comprised ten items about health and eating behavior. Responses are based on a four-point Likert scale where 1 = never, 2 = rarely, 3 = often, and 4 = always. The internal consistency of the original scale was 0.83. This scale was validated in Spanish and the psychometric properties were deemed adequate. The internal consistency for the Spanish version was 0.841. The range of scores was between 10 and 40 points. The cut-off point was ≥30 points in order to label people with orthorexia nervosa (ON) [25].

#### 2.3.3. The ORTO-11-ES Questionnaire

The ORTO-15 questionnaire was originally developed in Italian [9]. This tool consists of 15 self-report multiple choice items using a 4-point Likert-type scale (always, often, sometimes, never) to measure three underlying factors related to eating behavior: Cognitive–rational (items 1,5, 6, 11, 12, and 14), clinical (items 3, 7, 8, 9, and 15), and emotional aspects (items 2, 4, 10, and 13). It is used to investigate obsessive behavior related to the selection and preparation of food, habits of food consumption, and attitudes toward healthy food. The lower the score, the higher the indication of a behavior or attitude related to orthorexia. The Italian group [9] suggested a cut-off score of 40 points, whereby scores below this figure indicated ON-related behavior. For the present study, we used the ORTO-11-ES [14] as a tool for assessing ON. This tool is based on a structure of three factors for the abbreviated 11-item version and demonstrates an appropriate internal consistency (Cronbach’s alpha = 0.80). Furthermore, the test demonstrated a good predictive capacity for a threshold value of <25 (79.5% effectiveness, 75% sensitivity, and 79.6% specificity).

#### 2.3.4. The Eating Disorder Inventory-EDI-2-Spanish Version

The Eating Disorder Inventory (EDI-2) is a self-reported 91-item questionnaire, answered on a six-point Likert-Type-Scale, using a three-point system where “sometimes”, “rarely”, and “never”, are assigned zeros, while “often”, “usually” and “always” are assigned a score of 1, 2, or 3, respectively. The questionnaire is used to assess eating-disorder symptoms, attitudes, and behaviors. It contains 11 subscales: Drive for thinness, body satisfaction, bulimia, effectiveness, perfectionism, interpersonal disruption, interceptive awareness, maturity fears, asceticism, impulse regulation, and social insecurity. The sub-scale scores can be calculated by simply adding the scores of all the items of each specific sub-scale. The EDI-2 total score ranges from 91 to 546. We used a Spanish version of the scale validated by Corral, Pereña, and Seis-dedos in 1999, which showed an internal consistency of 0.83–0.92 [31].

The EDI-2 is a widely used tool in Spain. The validity of this tool has been proven for its accuracy in the detection and diagnosis of the risk of developing eating disorders among the Spanish population [32]. In this study, the EDI-2 was used based on its good psychometric proprieties in both clinical settings and non-clinical samples [33], as well as the ability to evaluate different dimensions [34].

### 2.4. Analysis

The statistical analyses were applied with SPSS 25.0 (IBM, Armonk, NY, USA). The χ^2^ test was developed with gender, Body Mass Index (BMI), type of studies, and DOS and ORTO. BMI was structured in several levels: <18.5 was underweight, between 18.5 and 24.9 was normal, between 25.0 and 29.9 was overweight, and finally, ≥30 was obese. Crosstabs were performed with the percentage of each cell.

Regarding the DOS, the authors applied a cutoff score of 30 points. In the case of the ORTO-11-ES, the cutoff score was less than 25 points [14]. The two sample *t*-test is a statistical procedure in order to compare the means in two independent groups. In this study, the dependent variables were the subscales of EDI-2. The Kolmogorov Smirnov test showed that all dependent variables fit a normal distribution (*p* > 0.05). The variables of DOS and ORTO were calculated and included the two sample *t*-test as independent variables. The relationships between the variables were statistically significant (*p* value < 0.05).

## 3. Results

### 3.1. Prevalence of Orthorexia with DOS-ES and ORTO-11-ES

The average score of the DOS scale was 17.52 (SD = 5.16) with a range of 10–38. The cutoff point for this scale was >30, or more, according to the 95th percentile. In this sense, the results showed that only 10.5% were participants with ON while 89.4% displayed no prevalence of ON. On the other hand, according to the ORTO, 25.2% of the sample showed indications of orthorexia nervosa, with the remaining 74.8% showing no sign of orthorexia nervosa. The score average of ORTO was 28.44 (SD = 5.67) with a range of 12–44 (see Table 1).

### 3.2. Differences between Orthorexia and Subscales of EDI-2

Table 2 shows the Analysis of Variance (ANOVA) between subscales of EDI-2 and the prevalence according to the DOS. In this sense, there were significant differences in the following subscales: Drive for thinness (F(2, 491) = 16.90, *p* < 0.01), body dissatisfaction (F(2, 491 = 3.50, *p* < 0.05), perfectionism (F(2, 491 = 4.83, *p* < 0.01), interceptive awareness (F(2, 491 = 3.83, *p* < 0.05), and asceticism (F(2, 491 = 13.54, *p* < 0.01).

Table 3 shows the two-sample *t*-test between subscales of EDI-2 and the prevalence according to the ORTO. In this sense, there were significant differences in the following subscales: Drive for thinness (*t*(1, 489) = 10.24, *p* < 0.01), bulimia (*t*(1, 489) = 4.19, *p* < 0.01), body dissatisfaction (*t*(1, 489) = 6.61, *p* < 0.01), perfectionism (*t*(1, 490) = 2.92, *p* < 0.01), effectiveness (*t*(1, 489) = 2.74, *p* < 0.01), interceptive awareness (*t*(1, 489) = 4.84, *p* < 0.01), asceticism (*t*(1, 489) = 4.93, *p* < 0.01), and impulse regulation (*t*(1, 489) = 3.13, *p* < 0.01). Obviously, in all cases the higher numbers related to the people with ON.

### 3.3. Relationships between Orthorexia and EDI-2 Subscales

Table 4 shows significant correlations with a confidence level of *p* < 0.01 and *p* < 0.05 between ORTO, DOS, and EDI-2 subscales. For instance, the correlation between ORTO and DOS was (*r* = −0.610, *p* < 0.01). Moreover, the Cronbach’s alpha of the ORTO scale was α = 0.841; for the DOS it was α = 0.791. The remaining significant correlations between the ORTO, DOS, and EDI-2 subscales are displayed in Table 4.

## 4. Discussion

Researchers and professionals are faced with situations where they are required to choose between several potentially viable scales which are designed for measuring a certain construct. At times, it is difficult to determine, in any field, which of the scales is the most useful.

In general, our findings regarding the prevalence of ON, based on the two questionnaires used in our sample, agree with the scientific literature published to date [13,24,30,35,36], albeit with very mixed results according to the tool used. However, the distinction is that this study has been administrated to the same sample. The prevalence detected using the ORTHO-11-ES tool was noticeably higher than that detected by the DOS-ES regarding the risk of suffering orthorexia, although the DOS-ES tool enabled us to establish a varying prevalence according to whether a risk of suffering orthorexia [24] was detected. The DOS-ES therefore differed when compared to the widely used ORTO-11-ES, in that it offered a possibility of distinguishing two groups of behaviors in relation to this pathology. However, even when both values were added, i.e., those at risk and those with a prevalence of ON, the end result remained lower than that provided by the ORTO-11-ES.

One of the causes of this disparity regarding the resulting prevalence values may be due to the psychometric properties of the ORTO-15 [9] in the different validations, as these vary depending on the country or respective translation used. The original ORTHO-15 [9] questionnaire decreased by an important number of items, changing the factorial structure in the different versions as well as the originally established cut-off point, whereas, in the DOS [24], both the 10 item structure and the established cut-off point were fully maintained by the original authors of the questionnaire. In favor of the ORTO-15 [9], we can argue that it was one of the first tools which, from its inception, attempted to provide insight into this pathology, while raising awareness in society and among scientists regarding the incipient development of ON in our increasingly obsessed 21st century culture of healthy eating. In addition, it is important to add that neither at that time nor to date has there been a consensus in the diagnostic criteria; therefore, the suitability of including certain items, or not, is even more complicated [1,2,4].

It is important to highlight that the sociocultural differences appear as part of the discussion in many validations of these tools, especially with regard to the ORTO-15 [37,38] and, more recently, the DOS. Societies exist where the cultural and/or religious beliefs mean that these populations have a well-established cultural relationship with food. In this sense, it is important to clarify that the problem in the detection of the risk of ON and in relation to different cultures is precisely in this fine line that separates the desire to follow a healthy diet or lifestyle from displaying an obsession for the same, which is considered a pathological approach toward eating. There are cultures which have always highly valued healthy eating as being the basis of their way of living, whereas other cultures have very different ingrained ideas regarding the subject. In China, for example, a study developed by Jinbo He et al. showed a prevalence of 7.8% using a validation of the DOS, with the authors assuming that the risk of ON in this population may be greater due to the fact that it is a culture with over 100 years of history regarding the maintenance of a healthy diet [13].

In contrast with the ORTO, the DOS is relatively new and its use is still limited to populations where the validation and translation of the same has been performed. However, both in the construction and in the psychometric properties, to date, improved results have been reported in the validation of this new tool [13,24]. These positive results should encourage and inspire researchers to continue this line of study in order to understand a behavior that is still undefined. It is important to remember that the tools and research used to evaluate behaviors should also support incorporating and/or evaluating the physical responses that occur in ON, which could contribute toward a differential diagnosis [39,40].

Another association which has been studied within the reported prevalence is the possible correlation of ON with gender. In our study, we observed that women are more likely to suffer ON, which correlates with other studies [12,29,41,42,43]. In contrast, other articles have noted a greater prevalence among men [13,21,35,44], whereas others have found no relationship [42,45,46,47]. All these results have led to the understanding that ON does not distinguish between gender. The stereotypes of health and beauty in the 21st century are continuously changing; the mass media bombard us and influence us in everyday decisions, including how we should eat, the way we dress, and how we take care of our body. This media onslaught does not discriminate between men and women regarding influencing lifestyles.

The present findings also reveal that the association with BMI was very low and statistically non-significant with both questionnaires, which is in line with most studies [13,23,24,39,48]. The greatest concern of these individuals is to maintain a healthy body, within a context of health in all its forms. Some authors have suggested that the onset of ON may be due, at times, to the beginning of diets to lose weight, meaning that when these are maintained over time, it can trigger an obsession for healthy eating in the individual [49]. The fact that a negative correlation exists between the BMI and the risk of ON has been perceived by some authors as a definitive fact for establishing this lack of relationship between ON and eating disorders (EDs) [24]. However, other authors have also found a relationship between ON with a low BMI. In a study by Gezer and Kabara [50], the risk of ON was found to be significantly greater in those with a low BMI (≤18.5 kg/m^2^) compared with other groups; however, this data on its own should not be considered a definitive criteria for establishing a clear diagnosis, for example of anorexia nervosa (AN).

Both scales have been found to correlate with different dimensions of the EDI-2. Among the dimensions that have not been correlated with any of the questionnaires are “interpersonal disruption and maturity fears”, both of which are dimensions which have less in common with the ON construct. The desire to return to the safety of infancy or the desire to publicly hide attitudes or thoughts related to a person’s eating behavior are more related to other EDs. Besides, in the case of ORTO, this is not correlated with “social insecurity”, which is a dimension which, besides being considered an essential trait when studying EDs, certainly is considered as being an essential trait in the assessment of ON with regard to the social isolation that it causes. In the English validation of the DOS by Chard et al. [24], all scores are minimal for these two dimensions and no correlation was found with DOS. Barthel et al. [11] only used three subscales of the EDI-2 and found correlations, although, at the same time, they used the most discussed dimensions regarding a possible relationship between other EDs and ON. These relationships have been widely discussed by a number of authors [1,2,3,4,34] with the obsession for thinness being the feature most highlighted by different researchers on this subject [20,34]. The items associated with this dimension attempt to encompass the concern that the individual has for weight and diets, considering this as one of the psychological nuclei present in EDs. In the criteria established by Barthel et al. in 2015, in relation to the diagnosis of ON, weight loss or being underweight is, indeed, one of the defining criteria of ON, with the exception that this excessive concern about weight does not always dominate the syndrome. In these cases it is recommended to assess another ED diagnosis [3]. If we consider that many authors consider ON as a subtype of AN [51], and having clearly established the relationship between obsession for thinness and anorexia or ON, the key is that the orthorexia questionnaires must provide the key to shift the balance more toward the well-established discussion between quality versus quantity of food [5]. The use of the EDI-2 is more widespread in patients with a confirmed clinical diagnosis, therefore, the use of this in patients who are lacking this clinical aspect has led to a clarification of whether some of the dimensions of certain EDs may be predictors of behavior in those that show a risk of suffering ON [52]. Therefore, further studies are required to confirm what some authors are suggesting. In this sense, the ORTHO-15 is the tool that has received most criticism to date, precisely because of the inability to distinguish between a healthy lifestyle, including lifestyles which have dietary restrictions, and behaviors that drive ON. In the case of the DOS, this tool does not demonstrate these imperfections in its psychometric properties. Furthermore, considering there are fewer studies using this tool, to date, no study has demonstrated that this may be the ideal instrument for performing a definitive screening in this population.

The field of orthorexia nervosa is rather new and it is important to have a good instrument which is able to measure both the healthy aspects (self-care) and unhealthy aspects of orthorexia nervosa. New studies along these lines support the existence of a new entity named Healthy Orthorexia (HO), compared to ON. These entities are not considered a continuum, rather as related, but differentiated, entities, where the dimensions related to psychopathological aspects are only present in ON and not in HO. A new questionnaire has been developed by researchers in an attempt to demonstrate the differences between people who adopt a healthy diet without evidence of obsessive behavior or any type of negative impact on their health and those who, as the definition of ON states, take this obsession to extremes which may damage their health [53,54].

The limitations of this study are mainly based on the impossibility of generalizing these results, as this study was performed on Spanish university students. A larger number of studies are required to understand the possible differences in the prevalence rates, not only regarding the application of different questionnaires but also the application of these findings to different populations, including those individuals with different dietary habits. Additionally, it is important to underline the fact that the participants used self-administered questionnaires, which may have influenced the objectivity of certain data, such as self-reported height and weight; this means the calculated BMIs based on this data may contain errors, as shown by other authors [52]. Furthermore, as this study was conducted on a university population, with their own distinct characteristics, it is impossible to extrapolate these data to the general population.

## 5. Conclusions

The different prevalence rates found with both tools lead us to affirm that the development of a comprehensive, sensitive, and valid questionnaire for assessing the symptoms of orthorexia should be supported by further studies with clinical patients, where observation and/or possible treatment measures may provide information regarding the pattern of the illness. It is important to consider that questionnaires are valuable tools for screening patients within a clinical environment, however, this does not replace the clinician’s diagnostic criteria. In this sense, research must advance in both ways, i.e., by screening large populations who are susceptible to be at risk of ON, where health measures would be valuable for prevention and in clinical practice.

## Figures and Tables

**Table 1:** Prevalence of orthorexia according to gender, Body Mass Index (BMI), and type of studies between the Düsseldorf Orthorexia Scale (DOS) and the test for the diagnosis of Orthorexia (ORTO).

Variables		DOS (%)	ORTO (%)	χ^2^ Test (DOS)	*p* Value (DOS)	χ^2^ Test (ORTO)	*p* Value (ORTO)
Gender	Male	4.1	6.7	1.14	0.274	18.12	0.00
	Female	6.5	18.5				
BMI	Underweight	1.0	1.4	0.623	0.891	4.18	0.312
	Normal	7.6	17.9				
	Overweight	1.6	4.5				
	Obese	0.2	1.4				
Studies	Engineering	3.6	6.7	0.387	0.534	6.40	0.04
	Health Sciences	6.9	18.5				

**Table 2:** Analysis of Variance between (DOS) and subscales of the Eating disorder Inventory (EDI-2).

Subscales of the Eating Disorder Inventory	DOS	N	M	SD	F
Drive for thinness	Up to 24	440	13.91	8.31	16.90 *
	25–29	41	20.22	8.03	
	Greater than or equal to 30	11	23.27	8.78	
Body dissatisfaction	Up to 24	440	17.09	9.87	3.50 **
	25–29	41	20.59	11.21	
	Greater than or equal to 30	11	22.27	14.63	
Perfectionism	Up to 24	440	14.26	5.83	4.83 *
	25–29	41	16.46	5.46	
	Greater than or equal to 30	11	18.09	5.37	
Interceptive awareness	Up to 24	440	16.85	8.63	3.83 **
	25–29	41	19.44	9.20	
	Greater than or equal to 30	11	22.55	6.74	
Asceticism	Up to 24	440	12.53	5.41	13.54 *
	25–29	41	16.27	6.02	
	Greater than or equal to 30	11	18.09	6.70	

* *p* < 0.01; ** *p* < 0.05.

**Table 3:** Two sample *t*-test for differences of means (ORTO) between subscales of EDI-2.

Subscales of the Eating disorder Inventory	ORTO *	N	M	SD	*t* test
Drive for thinness	with ON	124	20.79	7.82	*t*(1, 489) = 10.11, *p* < 0.01
	without ON	367	12.60	7.77	
Bulimia	with ON	124	10.95	6.79	*t*(1, 489) = 4.49, *p* < 0.01
	without ON	367	8.25	5.41	
Body dissatisfaction	with ON	124	22.65	10.81	*t*(1, 489) = 6.80, *p* < 0.01
	without ON	367	15.79	9.31	
Perfectionism	with ON	124	15.41	6.39	*t*(1, 490) = 2.92, *p* < 0.01
	without ON	367	14.25	5.62	
Effectiveness	with ON	124	15.57	9.31	*t*(1, 489) = 1.92, *p* < 0.01
	without ON	367	13.32	7.97	
Interceptive awareness	with ON	124	20.27	8.91	*t*(1, 489) =4.60, *p* < 0.01
	without ON	367	16.20	8.36	
Asceticism	with ON	124	14.74	5.93	*t*(1, 489) = 4.11, *p* < 0.01
	without ON	367	12.37	5.41	
Impulse regulation	with ON	124	17.10	9.33	*t*(1, 489) = 3.2.38, *p* < 0.05
	without ON	367	14.91	8.70	

* ON = Orthorexia nervosa.

**Table 4:** Correlation matrix between ORTO, DOS, and EDI-2 subscales.

Correlation	1	2	3	4	5	6	7	8	9	10	11	12
1. ORTO												
2. DOS	−0.610 **											
3. Drive for thinness	−0.594 **	0.500 **										
4. Bulimia	−0.243 **	0.195 **	0.347 **									
5. Body dissatisfaction	−0.390 **	0.288 **	0.719 **	0.384 **								
6. Perfectionism	−0.162 **	0.176 **	0.091 *	0.267 **	0.011							
7. Effectiveness	−0.165 **	0.160 **	0.336 **	0.361 **	0.460 **	0.185 **						
8. Interpersonal disruption	−0.044	0.029	0.076	0.223 **	0.179 **	0.162 **	0.506 **					
9. Interceptive awareness	−0.291 **	0.237 **	0.378 **	0.539 **	0.395 **	0.313 **	0.609 **	0.429 **				
10. Maturity fears	−0.040	−0.007	0.102 *	0.172 **	0.142 **	0.124 **	0.304 **	0.139 **	0.333 **			
11. Asceticism	−0.262 **	0.331 **	0.439 **	0.478 **	0.412 **	0.321 **	0.543 **	0.338 **	0.625 **	0.192 **		
12. Impulse regulation	−0.170 **	0.166 **	0.222 **	0.464 **	0.221 **	0.310 **	0.466 **	0.307 **	0.623 **	0.251 **	0.590 **	
13. Social insecurity	−0.075	0.092 *	0.160 **	0.263 **	0.307 **	0.155 **	0.672 **	0.684 **	0.453 **	0.210 **	0.467 **	0.445 **

** *p* < 0.01, * *p* < 0.05.
